# Long-term effects of fine particulate matter exposure on the progression of arterial stiffness

**DOI:** 10.1186/s12940-020-00688-6

**Published:** 2021-01-06

**Authors:** Dianqin Sun, Yue Liu, Jie Zhang, Jia Liu, Zhiyuan Wu, Mengyang Liu, Xia Li, Xiuhua Guo, Lixin Tao

**Affiliations:** 1 School of Public Health, Capital Medical University, Beijing, 100069 China; 2 Beijing Municipal Key Laboratory of Clinical Epidemiology, Beijing, 100069 China; 3 National Cancer Center/National Clinical Research Center for Cancer/Cancer Hospital, Chinese Academy of Medical Sciences and Peking Union Medical College, Beijing, 100021 China; 4 Department of Mathematics and Statistics, La Trobe University, Melbourne, Australia

**Keywords:** Fine particulate matter, Arterial stiffness, Ankle-brachial index, Brachial-ankle pulse wave velocity

## Abstract

**Background:**

Prior studies have investigated the association of PM_2.5_ exposure with arterial stiffness measured by ankle-brachial index (ABI) and brachial-ankle pulse wave velocity (baPWV), of which conclusions are inconsistent. Moreover, limited evidence is available on the contributory role of PM_2.5_ exposure on the arterial stiffness index.

**Methods:**

We used the population data from the Beijing Health Management Cohort and conducted a longitudinal analysis. The annual average concentration of PM_2.5_ for 35 air pollutant monitoring sites in Beijing from 2014 to 2018 was used to estimate individual exposure by different interpolation methods. Multivariate logistic regression and linear regression were conducted to assess the association of annual average PM_2.5_ concentration with the incidence of higher baPWV, the progression of ABI, and baPWV, respectively.

**Results:**

The association between PM_2.5_ exposure and incidence of higher baPWV was not significant (OR = 1.11, 95% CI: 0.82–1.50, *P* = 0.497). There was − 0.16% (95% CI: − 0.43-0.11%) decrease in ABI annually and 1.04% (95% CI: 0.72–1.37%) increase in baPWV annually with each increment of 10 μg/m^3^ average PM_2.5_ concentration.

**Conclusions:**

Long-term exposure to PM_2.5_ was associated with the progression of arterial stiffness in Beijing. This study suggests that improvement of air quality may help to prevent arterial stiffness.

**Supplementary Information:**

The online version contains supplementary material available at 10.1186/s12940-020-00688-6.

## Background

Recent decades have witnessed the rapid growth of the Chinese economy, unfortunately, along with severe fine particulate matter pollution, especially in north China [1]. Fine particulate matter, known as PM_2.5_, has been regarded as one of the culprits in cardiovascular diseases [2, 3]. It has been reported that increased long-term exposure to PM_2.5_ is associated with elevated cardiovascular morbidity and mortality [4]. The potential mechanism behind the association has become a subject of research. The effect of PM_2.5_ on arterial stiffness, one of the pathways to severe cardiovascular diseases, has attracted more and more attention [5–7].

Increased arterial stiffness, a measure of vascular dysfunction, is highly relevant to the atherosclerosis process and is also seen as an independent risk factor for cardiovascular events [8]. Ankle-brachial index (ABI) and pulse wave velocity (PWV) are two sensitive markers for arterial stiffness and easy to use. ABI is measured as the ratio of systolic blood pressure at the ankle to that at the brachial artery [9]. The low ABI is used to diagnose peripheral artery disease in clinical practice, while the high ABI may indicate non-compressible arteries and arterial calcification. PWV is the most widely used measure of arterial stiffness, which was calculated by dividing the distance between the arteries’ two points with the corresponding pressure wave transit time. Various types of PWV measurements were applied in clinical and academic fields, among which carotid-femoral PWV (cfPWV) and brachial-ankle PWV (baPWV) are the most widely used. Brachial-ankle PWV is strongly associated with cfPWV [10], the gold standard for measuring central arterial stiffness [11], but baPWV measurement is more conveniently used and popular in Asian countries [12]. Previous studies have investigated the association of PM_2.5_ exposure with baPWV and ABI [5, 13–15], of which conclusions are inconsistent. A cross-sectional study reported the relationship between PM_2.5_ exposure and ABI [13], which was not observed in other studies [14, 16].

Furthermore, few were conducted to investigate the long-term effects in areas with relatively more severe PM_2.5_ pollution in China. The nonlinear relationship between air pollution and health effects may indicate that evidence from other regions may not be suitable to the population in areas with high air pollution in Beijing, China. Moreover, it was reported that racial differences might exist in air pollutants’ cardiovascular effect [17].

Therefore, in this study, we used the population data from the Beijing Health Management Cohort (BHMC) and conducted a longitudinal analysis to investigate the long-term effects of PM_2.5_ exposure on the change of arterial stiffness markers, including ABI and baPWV.

## Methods

### Study design and population

We conducted this study using data from the BHMC, a large dynamic cohort aiming fixed working environment population in Beijing, of which design has been described in previous studies [18]. Participants in BHMC underwent a comprehensive health examination every year or every 2 years. Since our aim was to estimates the effects of PM_2.5_ exposure on the progression of ABI and baPWV, we retrieved data of participants who underwent at least two examinations of ABI and baPWV (*n* = 4789) during 2014–2018. The first and last measurements were used. Then those participants with abnormal ABI (ABI < 0.9 or ABI > 1.4) or baPWV (baPWV > 1800 cm/s) at first measurement were excluded (*n* = 838). Finally, 3951 participants were included in the study. All participants provided written informed consent. This study was approved by the Ethics Committees of Capital Medical University (approval number: 2013SY26).

### Measures of arterial stiffness

ABI and baPWV were measured using an automatic arteriosclerosis analyzer. The ABI is measured as the ratio of systolic blood pressure at the ankle to that at the brachial artery. ABIs were calculated separately for both sides by the highest pressure of the ankle on the side divided by the highest brachial pressure on either side, and the minimum was used for analyses. According to previous studies [19–21], lower ABI, higher ABI and higher baPWV was respectively defined as ABI < 0.9, ABI > 1.4 and baPWV > 1800 cm/s. Besides, the progression of ABI and baPWV were assessed using the relative annual change (%) [22], which was calculated via the equation: $\frac{value\  at\  last\ measurement- value\  at\  first\ measurement}{follow- up\  year\times value\  at\  first\ measurement}$.

### PM_2.5_ exposure assessment

We collected daily average PM_2.5_ concentration data from 2014 to 2018 recorded by 35 monitoring sites from the Beijing Municipal Environmental Monitoring Center’s website (http://zx.bjmemc.com.cn/). The annual average concentration in each monitoring site was calculated. The empirical Bayesian Kriging method was applied to assess individual exposure based on the workplace address using ArcGIS 10.5 software [23]. Because PM_2.5_ data recorded by monitors were not available before 2014, pollution data from 2012 to 2013 was gained from PM_2.5_ Hindcast Database. PM_2.5_ Hindcast Database [24] provides annual concentrations of PM_2.5_ in a regular grid of 0.1° × 0.1°, developed and maintained by Tsinghua university based on Moderate Resolution Imaging Spectroradiometer (MODIS) satellite aerosol datasets. In addition, we also used the Inverse Distance Weighting (IDW) interpolation method and data from the nearest monitoring site to estimate individual exposure in the sensitivity analysis. According to prior studies [25, 26], the average value (μg/m^3^) from the baseline year to the last visit year was used as the estimated exposure. Because PM_2.5_ data from 2014 to 2016 were also available in PM_2.5_ Hindcast Database, we could conduct linear regression to test the correlation between data from PM_2.5_ Hindcast Database and those calculated based on records from monitoring sites during 2014 to 2016. The results indicated that data in PM_2.5_ Hindcast Database were strongly correlated with the estimates of the IDW interpolation method (Supplementary Table S1). However, data by the empirical Bayesian Kriging method tended to be higher, and data from the nearest monitoring site tended to be lower than estimates in PM_2.5_ Hindcast Database.

### Covariates assessment

In BHMC, trained medical personnel conducted the physical examination. Height and weight were measured, and body mass index (BMI) was calculated according to the formula: $\mathrm{BMI}=\frac{Weight\ (kg)}{Height\ {(m)}^2}$. Blood pressure was measured three times after participants were seated, and the average value was used. Fasting blood samples were collected. Triglycerides (TG), total cholesterol (TC), high-density lipoprotein cholesterol (HDL-C), low-density lipoprotein cholesterol (LDL-C), and fasting plasma glucose (FPG) were measured by enzymatic methods using a chemistry analyzer (Beckman LX 20, Beckman, Brea, CA, USA). In this study, FPG ≥ 100 mg/dL or using anti-diabetic drugs was defined as diabetes. Hypertension was defined as elevated blood pressure (systolic BP ≥ 130 mmHg and/or diastolic BP ≥ 85 mmHg) or antihypertensive drug treatment. Demographic data, including age, gender, education, smoking, alcohol use, and physical activities, were collected by self-administered questionnaires. We defined smoking as currently smoking and/or having smoked at least 100 cigarettes in one’s lifetime, drinking as having consumed alcohol 12 or more times in the last year. Physical activity was classified into three levels: low, moderate, and high intensity.

### Statistical analysis

Characteristics of participants were described with the mean (standard deviation, SD) for continuous variables with normal distribution or median (interquartile range, IQR) for those with skewed distribution and number (percentage) for categorical variables. Baseline characteristics were compared with *t* test, Wilcoxon rank test, or *χ*^2^ test, as appropriate. Because the number of participants with abnormal ABI incidence was too small (*n* = 4 for ABI > 1.4, *n* = 14 for ABI < 0.9), we did not investigate the association of annual average PM_2.5_ concentration with abnormal ABI incidence. We performed logistic regression to assess the association between annual average PM_2.5_ concentration and the incidence of high baPWV. The odds ratio (OR) and its 95% Confident interval (CI) were calculated. Then restricted cubic spline was used to depict the dose-response relationship and test whether the nonlinear association exists. Multivariate linear regression was conducted to investigate the relationship of annual average PM_2.5_ concentration with the progression of ABI and baPWV. We used a step-by-step method to adjust confounding factors in the multivariate models, where the various number of participants were included due to missingness of covariates: (1) crude model (*n* = 3951); (2) model controlling for age and gender (n = 3951); (3a) model additionally adjusting for BMI, HDL-C, LDL-C, TG, systolic BP, and diabetes (*n* = 3684); (3b) model adjusting for the same set of covariates with model 3a in the same population with model 4 (*n* = 1128); (4) model additionally controlling for education, smoking, alcohol consumption and physical activity (n = 1128). Model 4 worked as our final model, and its results were mainly reported.

We also conducted subgroup analyses by hypertension status and age group (age > 55, age ≤55) to see which group is more sensitive to an increase in PM_2.5_ concentration. To test whether the results were robust, we conducted several sensitivity analyses. Multivariate logistic and linear regression was repeatedly conducted with different methods to assess exposure (inverse distance weighted, the nearest monitor method) and different cut-off values to define higher baPWV [27, 28] (1400 cm/s, 1700 cm/s).

Statistical analyses were executed by SAS version 9.3 (SAS Institute, Cary, NC, USA). Two-tailed *P* values < 0.05 were considered statistically significant.

## Results

The baseline characteristics of study participants are presented in Table 1. The total participants’ median age was 56 (IQR: 15) years, and 20.1% were women. Most participants have attained bachelor’s degrees, and more than half of the participants were diagnosed with hypertension. The mean BMI was 25.59 kg/m^2^, which fell within the overweight range.

**Table 1:** Baseline characteristics of the study participants (*n*=1128)

Characteristic	Distribution
annual average PM_2.5_ concentration (μg/m^3^) median (IQR)	76.44 (6.63)
Age (year) median (IQR)	56 (15)
Gender (female) *n*(%)	227 (20.12)
Education *n*(%)	
Middle school or below	40 (3.55)
High school	98 (8.69)
Bachelor’s degree	758 (67.20)
Master’s or above	232 (20.57)
Physical activity *n*(%)	
Low intensity	773 (68.53)
Moderate intensity	262 (23.23)
High intensity	93 (8.24)
Alcohol consumption *n*(%)	400 (35.46)
Cigarette smoking *n*(%)	302 (26.77)
HDL-C (mmol/L) median (IQR)	1.24 (0.42)
LDL-C (mmol/L) median (IQR)	3.03 (1.02)
BMI (kg/m^2^) mean (SD)	25.59 (3.06)
TG (mmol/L) median (IQR)	1.31 (0.92)
Hypertension *n*(%)	566 (50.18)
Diabetes *n*(%)	339 (30.05)

*IQR* Interquartile range, *HDL-C* High-density lipoprotein cholesterol, *LDL-C* Low-density lipoprotein cholesterol, *BMI* Body mass index, *SD* Standard error, *TG* Triglyceride

**Fig. 1:**
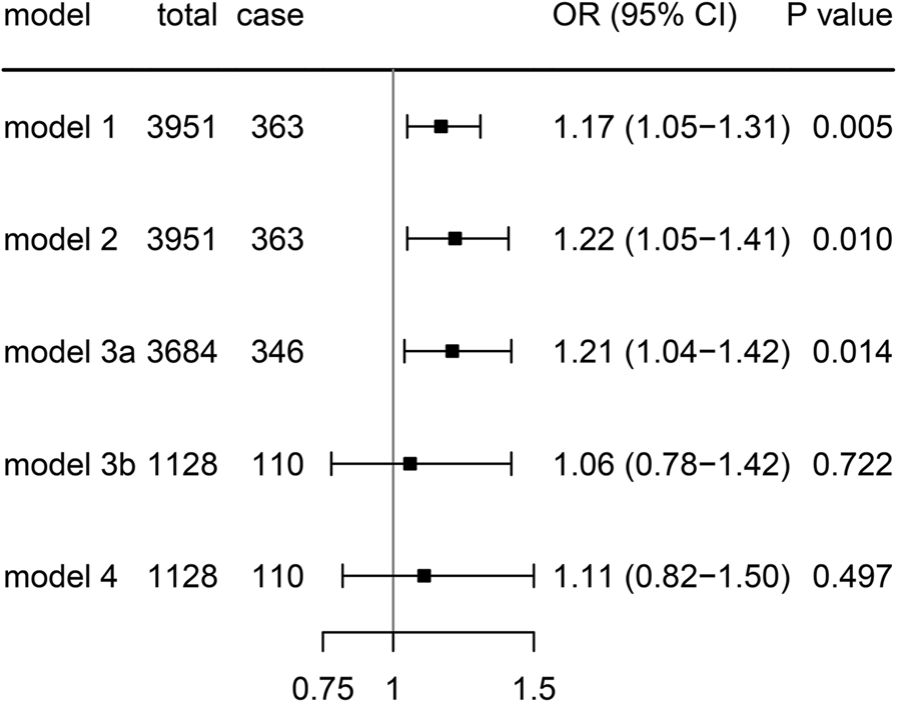
Association between increased 10 μg/m^3^ PM_2.5_ concentration and incidence of higher baPWV. Odds ratios indicated by black boxes were shown along with 95% confident intervals. Model 1: crude model; Model 2: additionally adjusted for age and gender; Model 3a: additionally adjusted for BMI, HDL-C, LDL-C, TG, systolic BP, and diabetes; Model 3b: adjusted for the same set of covariates with model 3a in the same population with model 4; Model 4: additionally controlled for education, smoking, alcohol consumption and physical activity

The effect of PM_2.5_ exposure on the incidence of higher baPWV is depicted in Fig. 1. In the crude model (model 1), PM_2.5_ was associated with incidence of higher baPWV (OR = 1.17, 95% CI: 1.05–1.31, *P* =0.005). After adjusting age and gender (model 2), participants had a 22% higher risk (OR = 1.22, 95% CI: 1.05–1.41, *P* =0.010) for each increment of 10 μg/m^3^ annual average PM_2.5_ concentration. The association was still significant in model 3a after controlling for BMI, HDL-C, LDL-C, TG, systolic BP, and diabetes. However, this association was not significant (OR = 1.11, 95% CI: 0.82–1.50, *P* = 0.497) after further adjusting for education, cigarette smoking, alcohol consumption, and physical activity (model 4). The association was not significant (*P* = 0.722) in model 3b, which was conducted adjusting for the same set of covariates with model 3a but in the same population as model 4. Besides, we noted a small effect estimate reduction from model 4 to models 3b, but not as large as model 3a, which indicated that model 4 might be mainly influenced by reduced sample size.

**Fig. 2:**
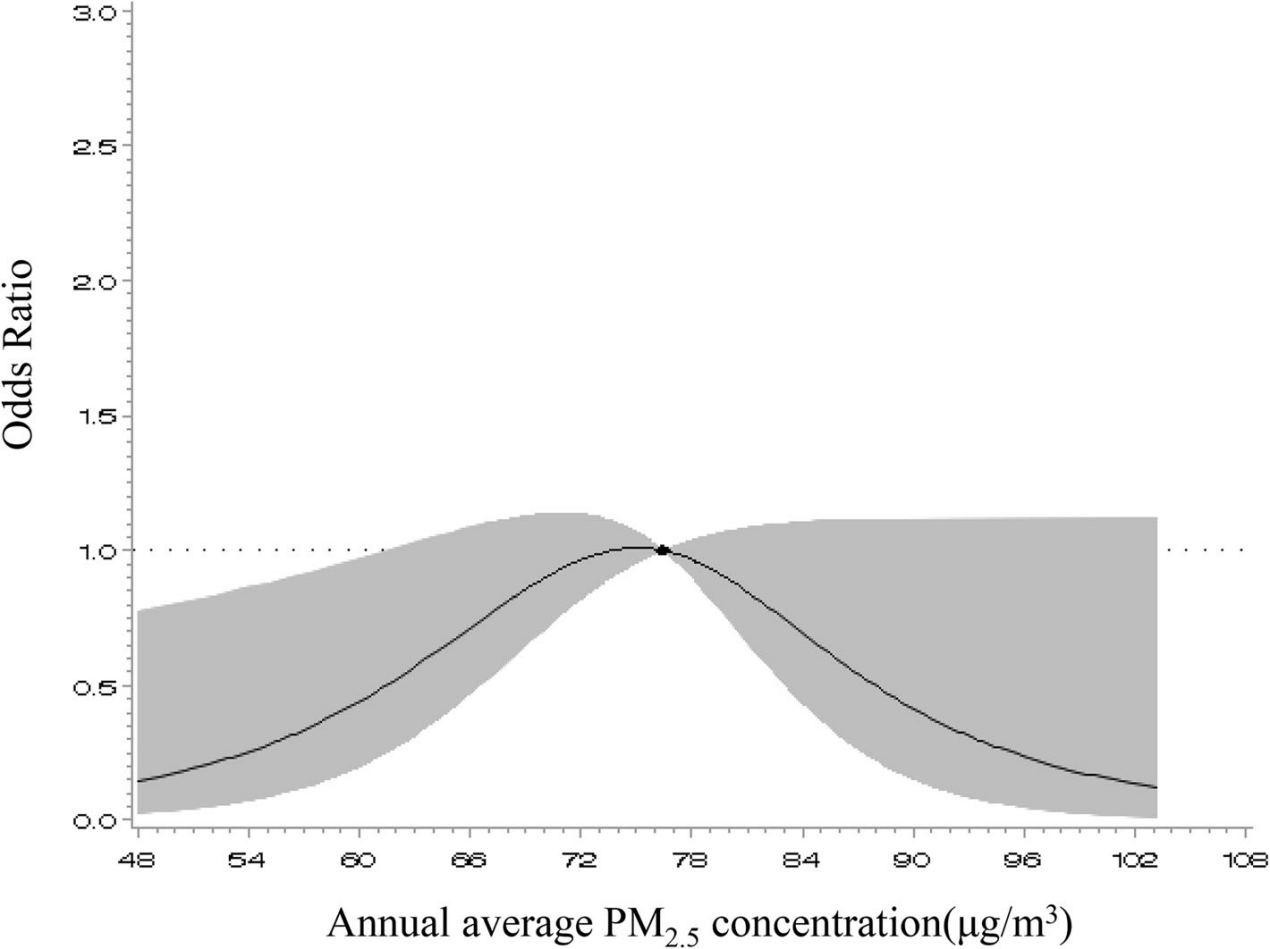
The relationship between annual average PM_2.5_ concentration and incidence of higher baPWV using adjusted restricted cubic spline. The reference value for each OR is the midpoint of the reference group (i.e., 76.44 μg/m^3^). The solid line represented estimated odds ratios and the cloud area indicated 95% CIs

A nonlinear curve was observed to associate the annual average PM_2.5_ concentration with the risk of higher baPWV in the analysis using restricted cubic splines (Fig. 2, *P* = 0.010 for the nonlinear test). There was a significant rise in risk with increasing annual average PM_2.5_ concentrations until the value approached approximately 60 μg/m^3^.

Associations of PM_2.5_ exposure with a relative annual change of ABI and baPWV are presented in Table 2. The relationship between annual average PM_2.5_ concentration and ABI progression was significantly negative in the crude model (model 1) but turned null after adjusting potential confounding factors (model 2-model 4). However, the effect of annual average PM_2.5_ concentration on relative annual change of baPWV was always significant from model 1 to model 4. There was a 1.04% (95% CI: 0.72–1.37%) gain in baPWV annually with each increment of 10 μg/m^3^ average PM_2.5_ concentration in the full adjusted model.

**Table 2:** Association between increased 10 μg/m^3^ PM_2.5_ concentration and relative annual change of ABI and baPWV

Outcome	Model	β (95% CI)	*P*
relative annual change of ABI	model 1	−0.00177 (− 0.00312,0.00042)	0.010
	model 2	−0.00127 (− 0.00262,0.00008)	0.065
	model 3a	−0.00077 (− 0.00216,0.00061)	0.274
	model 3b	−0.00166 (− 0.00438,0.00106)	0.232
	model 4	−0.00160 (− 0.00434,0.00114)	0.252
relative annual change of baPWV	model 1	0.00752 (0.00592,0.00912)	< 0.001
	model 2	0.00736 (0.00576,0.00896)	< 0.001
	model 3a	0.00801 (0.00636,0.00966)	< 0.001
	model 3b	0.01011 (0.00688,0.01334)	< 0.001
	model 4	0.01044 (0.00721,0.01367)	< 0.001

Model 1: crude model; Model 2: additionally adjusted for age and gender; Model 3a: additionally adjusted for BMI, HDL-C, LDL-C, TG, systolic BP, and diabetes; Model 3b: adjusted for the same set of covariates with model 3a in the same population with model 4; Model 4: additionally controlled for education, smoking, alcohol consumption, and physical activity.s

Subgroup analyses by age group (age≤55 and age > 55) showed interesting results (Supplementary Table S2 & Table S3). The association between the incidence of higher baPWV and PM_2.5_ exposure was marginally significant (*P* = 0.056) among participants younger than 55 years. Moreover, per 10 μg/m^3^ elevated PM_2.5_ concentration was associated with a 1.40% (95% CI: 0.94–1.86%) increase in baPWV annually among younger participants. However, this relationship was not observed among older individuals. Several sensitivity analyses were conducted to show that the results were robust (Supplementary Table S4 & Fig. S1). The estimates did not change apparently when different exposure assessment methods were used. We also applied different cut-off values to define higher baPWV and explored the relationship between the incidence of higher baPWV and PM_2.5_ exposure. It was worth noting that the association turned significant (*P* = 0.037) with 1700 cm/s as the threshold.

## Discussion

In this study, we conducted a longitudinal analysis to investigate the long-term effects of PM_2.5_ exposure on the progression of ABI and baPWV. We found a significantly positive association between PM_2.5_ and baPWV annual change rate in the cohort study but no association between PM_2.5_ and ABI annual change rate.

The association between PM_2.5_ exposure and incidence of higher baPWV was not significant in the final fully adjusted model 4. Due to the missingness of these covariates, the sample size in model 4 decreased apparently. To explore the reason for effects change, we conducted model 3b, which adjusted the same covariate with model 3a in the same population of model 4. The effects estimated in model 3b were similar to those estimated in model 4. It may warn that the confounding effect was small, and a reduced sample size brought false-negative results. Further studies with a larger sample are needed to verify the result.

Our results are partly consistent with previous studies. A study based on the Multi-Ethnic Study of Atherosclerosis cohort failed to find evidence of the relationship between PM_2.5_ exposure and ABI [14]. A study comprising 4814 men and women found that residential proximity to a major road was associated with ABI, but PM_2.5_ exposure was not [16]. However, a cross-sectional study [13] using quantile regression revealed a non-monotonic association between PM_2.5_ exposure and ABI. Simply speaking, participants with relatively low ABI tended to have an increased risk for lower ABI, but the ones with relatively high ABI tended to have higher ABI with increased PM_2.5_ exposure [13]. A study based on the Framingham Heart cohort did not observe the association between arterial stiffness measures and long-term levels of PM_2.5_ or short-term levels of PM_2.5_, where the arterial stiffness was assessed by cfPWV rather than baPWV [6]. However, the short-term effect of PM_2.5_ on baPWV was observed in a longitudinal study [15].

There are multiple biological mechanisms involved in the association between PM_2.5_ exposure and arterial stiffness. Inflammation could play a primary role in the process. Animal experiments demonstrated that exposure to PM_2.5_ intensified plaque progression in mice by inducing vascular inflammation [29]. It is well documented that inflammation could affect functional and structural changes in the arterial wall [30]. Several epidemiological studies [31, 32] also revealed a strong association between inflammatory markers and arterial stiffness. Besides, the effect of exposure to PM_2.5_ could be mediated through other plausible biologic mechanisms, including blood pressure and autonomic function [33].

According to our knowledge, this is the first study investigating the long-term effects of PM_2.5_ on the relative change of ABI and baPWV. Most previous studies were limited by only once measurement data. However, this study has several limitations. First, most published studies assessed personal PM_2.5_ exposure by each participant’s residential address rather than the workplace address used in this study. Second, many participants have the same average concentration of PM_2.5_ exposure (tied data); since enrolled participants in our study were organized by their companies. This might reduce the power of the statistical test. Third, pollution data sources were inconsistent from 2012 to 2018 because PM_2.5_ pollution data recorded by monitors were not available before 2014. Forth, the potential short-term effect of PM_2.5_ exposure on arterial stiffness was not adjusted in our analysis.

## Conclusions

In conclusion, there is a significantly positive association of PM_2.5_ with baPWV annual change rate, but no association between PM_2.5_ and ABI annual change rate was found. This study suggests that improvement of air quality may help to prevent arterial stiffness.

## Supplementary Information

**Additional file 1: Table S1.** Correlation between data from PM_2.5_ Hindcast Database and those calculated based on records from monitoring sites from 2014 to 2016. **Table S2.** Subgroup analysis for the association between increased 10 μg/m^3^ PM_2.5_ concentration and incidence of higher baPWV. **Table S3.** Subgroup analysis for the association between increased 10 μg/m^3^ PM_2.5_ concentration and relative annual change of ABI and baPWV. **Table S4.** Sensitivity analysis for the association between increased 10 μg/m^3^ PM_2.5_ concentration and relative annual change of ABI and baPWV. **Fig. S1.** Sensitivity analysis for the association between increased 10 μg/m^3^ PM_2.5_ concentration and incidence of higher baPWV. Odds ratios indicated by black boxes were shown along with 95% confident intervals.

## Data Availability

The datasets used and/or analyzed during the current study are available from the corresponding author on reasonable request.

## Abbreviations

ABI: Ankle-brachial index
baPWV: Brachial-ankle pulse wave velocity
OR: Odds ratio
BHMC: Beijing Health Management Cohort
BMI: Body mass index
TG: Triglycerides
TC: Total cholesterol
HDL-C: High-density lipoprotein cholesterol
LDL-C: Low-density lipoprotein cholesterol
FPG: Fasting plasma glucose
SD: Standard deviation
IQR: Interquartile range
CFPWV: Carotid-femoral pulse-wave velocity
