# Supplementary material for: Long-term effects of fine particulate matter exposure on the progression of arterial stiffness
Source: Environ Health. 2021 Jan 6;20:2. doi: 10.1186/s12940-020-00688-6 (PMC7789369; doi:10.1186/s12940-020-00688-6)
Supplement: Supplementary file 1 — Additional file 1: Table S1. Correlation between data from PM2.5 Hindcast Database and those calculated based on records from monitoring sites from 2014 to 2016. Table S2. Subgroup analysis for the association between increased 10 μg/m3 PM2.5 concentration and incidence of higher baPWV. Table S3. Subgroup analysis for the association between increased 10 μg/m3 PM2.5 concentration and relative annual change of ABI and baPWV. Table S4. Sensitivity analysis for the association between increased 10 μg/m3 PM2.5 concentration and relative annual change of ABI and baPWV. Fig. S1. Sensitivity analysis for the association between increased 10 μg/m3 PM2.5 concentration and incidence of higher baPWV. Odds ratios indicated by black boxes were shown along with 95% confident intervals. [file 12940_2020_688_MOESM1_ESM.docx]

Table S1. Correlation between data from PM_2.5_ Hindcast Database and those calculated based on records from monitoring sites from 2014 to 2016

| Year | Exposure assessment | β (95% CI) | R^2^ |
| --- | --- | --- | --- |
| 2014 | Empirical Bayesian Kriging | 1.02 (1.00,1.03) | 0.80 |
|  | Inverse Distance Weighted | 1.01 (1.00,1.02) | 0.88 |
|  | the Nearest Monitor | 0.75 (0.73,0.76) | 0.72 |
| 2015 | Empirical Bayesian Kriging | 1.58 (1.55,1.61) | 0.69 |
|  | Inverse Distance Weighted | 1.05(1.04,1.06) | 0.94 |
|  | the Nearest Monitor | 0.87(0.86,0.88) | 0.87 |
| 2016 | Empirical Bayesian Kriging | 1.52(1.50,1.55) | 0.73 |
|  | Inverse Distance Weighted | 0.96(0.95,0.97) | 0.92 |
|  | the Nearest Monitor | 0.81(0.80,0.82) | 0.86 |

Table S2. Subgroup analysis for the association between increased 10 μg/m^3^ PM_2.5_ concentration and incidence of higher baPWV.

| Variable | Total | Case | OR (95% CI) | *P* |
| --- | --- | --- | --- | --- |
| Hypertension |  |  |  |  |
| Yes | 566 | 84 | 1.14 (0.80,1.62) | 0.472 |
| No | 562 | 26 | 1.16 (0.64,2.10) | 0.624 |
| Age group |  |  |  |  |
| Age≤55 | 546 | 6 | 4.68 (0.96,22.8) | 0.056 |
| Age＞55 | 576 | 104 | 1.01 (0.72,1.41) | 0.957 |

Table S3. Subgroup analysis for the association between increased 10 μg/m^3^ PM_2.5_ concentration and relative annual change of ABI and baPWV

| Outcome | Subgroup | β (95% CI) | *P* |
| --- | --- | --- | --- |
| relative annual change of ABI | Hypertension-Yes | 0.00027 (-0.00338,0.00391) | 0.886 |
|  | Hypertension-No | -0.00342 (-0.00750,0.00066) | 0.100 |
|  | Age≤55 | 0.00059 (-0.00351,0.00468) | 0.778 |
|  | Age＞55 | -0.00647 (-0.00992,-0.00302) | <0.001 |
| relative annual change of baPWV | Hypertension-Yes | 0.00795 (0.00317,0.01273) | 0.001 |
|  | Hypertension-No | 0.01359 (0.00922,0.01796) | <0.001 |
|  | Age≤55 | 0.01401 (0.00938,0.01864) | <0.001 |
|  | Age＞55 | 0.00138 (-0.00323,0.00598) | 0.558 |

Table S4. Sensitivity analysis for the association between increased 10 μg/m^3^ PM_2.5_ concentration and relative annual change of ABI and baPWV

| Outcome | Exposure assessment | β (95% CI) | *P* |
| --- | --- | --- | --- |
| relative annual change of ABI | Empirical Bayesian Kriging | -0.00160 (-0.00434,0.00114) | 0.252 |
|  | Inverse Distance Weighted | -0.00143 (-0.00431,0.00145) | 0.331 |
|  | the Nearest Monitor | -0.00127 (-0.00403,0.00149) | 0.368 |
| relative annual change of baPWV | Empirical Bayesian Kriging | 0.01044 (0.00721,0.01367) | <0.001 |
|  | Inverse Distance Weighted | 0.01086 (0.00745,0.01427) | <0.001 |
|  | the Nearest Monitor | 0.01011 (0.00684,0.01338) | <0.001 |


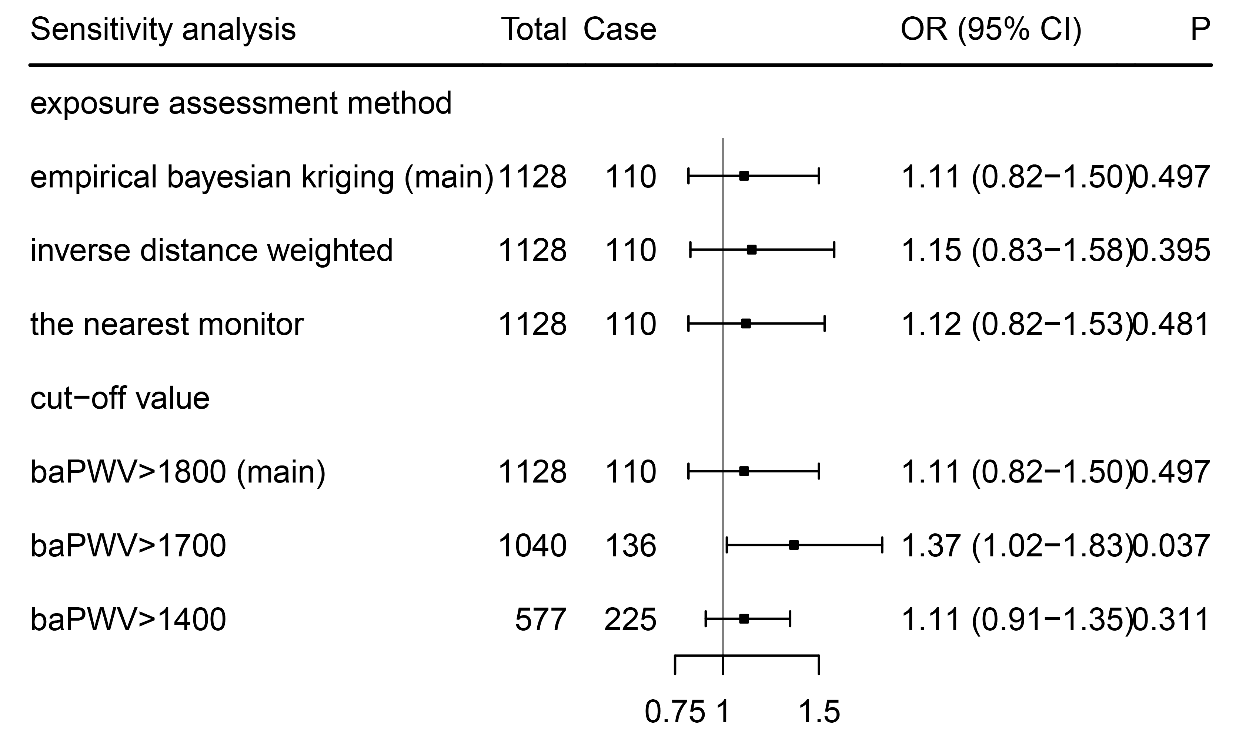


Figure S1. Sensitivity analysis for the association between increased 10 μg/m^3^ PM_2.5_ concentration and incidence of higher baPWV. Odds ratios indicated by black boxes were shown along with 95% confident intervals.
